# Susceptibility to eye diseases in relation to age and kidney failure among Taiwanese adults

**DOI:** 10.1186/s12877-024-04740-9

**Published:** 2024-02-19

**Authors:** Shin-Lin Chiu, Oswald Ndi Nfor, Chiu-Liang Chen, Disline Manli Tantoh, Wen Yu Lu, Pei-Hsin Chen, Yung-Po Liaw

**Affiliations:** 1 Department of Ophthalmology, Changhua Christian Hospital, 500 Changhua City, Taiwan; 2 College of Nursing and Health Sciences, Da-Yeh University, 515006 Changhua County, Taiwan; 3 Department of Post-Baccalaureate Medicine, College of Medicine, National Chung Hsing University, 40201 Taichung City, Taiwan; 4 Department of Public Health, Institute of Public Health, Chung Shan Medical University, No. 110, Sec. 1 Jianguo N. Rd, 40201 Taichung City, Taiwan; 5 Department of Orthopedics, Changhua Christian Hospital, 500 Changhua City, Taiwan; 6 Department of Nursing, Hungkuang University, 433 Taichung City, Taiwan; 7 Department of Medical Imaging, Chung Shan Medical University Hospital, 40201 Taichung City, Taiwan; 8 Medical Imaging and Big Data Center, Chung Shan Medical University Hospital, 40201 Taichung City, Taiwan

**Keywords:** Eye diseases, Cataract, Kidney failure, Age, Interaction, Taiwanese

## Abstract

**Background:**

The kidney and eyes share common pathways and are thought to be closely connected. Chronic kidney disease and major eye diseases, such as cataract and glaucoma, are strongly associated with age. However, further investigation is needed to understand the joint impact of age and kidney diseases on eye diseases. In this study, we assessed the risk of eye diseases in relation to age and kidney failure in Taiwanese adults.

**Methods:**

Our study included 127,561 cancer-free volunteers aged 30 to 70 years who participated in the Taiwan Biobank (TWB) project from 2008 to 2020. Information on the main exposures (kidney failure and age) and the outcome (eye diseases, including glaucoma, cataract, xerophthalmia, and retinal detachment) was collected through questionnaires.

**Results:**

In general, kidney failure and older age were independently associated with a higher risk of eye, particularly cataract and retinal detachment: prevalence odds ratio (POR); 95% confidence interval (CI) = 2.480; 1.635–3.761 for cataract and 3.885; 1.968–7.666 for retinal detachment. A significant interaction between kidney failure and age on cataract was observed (*p*-value = 0.0002). Age-stratified analysis revealed a higher risk of cataract among patients with kidney failure aged below 50 (POR = 6.534; 95% CI = 2.493–17.124) and between 50 and 60 years (POR = 3.957; 95%CI = 1.986–7.881). Combining kidney failure and age (reference: no kidney failure and age < 50 years), kidney failure in all age groups was associated with a higher risk of cataract. The PORs; 95% CIs were 10.725; 4.227–27.211 for patients below 50 years, 28.487; 14.270-56.866 for those aged 50–60 years, and 43.183; 24.434–72.824 for those > 60 years. Combining cataract and age (reference: no cataract and age < 50 years), patients below 50 years had the highest risk of kidney failure (POR; 95% CI = 9.510; 3.722–24.297).

**Conclusions:**

Our study suggests that age and kidney failure may jointly contribute to eye diseases, particularly cataract. The association between cataract and kidney failure could be bidirectional, especially in individuals below 50 years. This significant bidirectional relationship underscores the need for screening patients with cataract for kidney failure and vice versa, particularly in younger adults.

## Background

Various eye diseases, including glaucoma, cataract, xerophthalmia, and retinal detachment have been widely investigated. Glaucoma, a chronic progressive optic neuropathy, is the leading cause of irreversible blindness [1]. It is characterized by degenerating retinal ganglion cells (RGCs) which cause significant visual disability [2]. Cataract causes cloudy vision and is one of the leading causes of blindness globally [1]. Blindness due to cataracts affects approximately 15 million adults aged 50 years and older [1]. Several risk factors for cataract including age, smoking, UV-B light, and systemic diseases (diabetes, hypertension, and kidney failure) have been identified [3].

Kidney disease is a global public health concern. Kidney failure is an adverse outcome of chronic kidney disease [4]. Causes of kidney failure can be endogenous or exogenous. Some exogenous causes include cardiovascular diseases, diabetes, and lung/liver failure while endogenous causes include factors such as glomerular nephritis, polycystic kidney disease, renal fibrosis, tubular cell death, and kidney stones [5].

Epidemiological and pathological evidence shows that kidney disease may be linked to several eye diseases [6, 7]. For instance, patients with chronic kidney disease have a higher probability of suffering from age-related macular degeneration, diabetic retinopathy, glaucoma, and cataract [8]. Moreover, mechanisms such as atherosclerosis, vascular remodeling, endothelial dysfunction, inflammation, and oxidative stress underlie both chronic kidney disease and eye diseases [8–10].

Several studies have explored the links between eye and kidney diseases; however, a comprehensive understanding of how age influences susceptibility remains an area that requires focused investigation. The aging population in Taiwan has witnessed a surge in the prevalence of both eye diseases and kidney failure, prompting a critical examination of the relationship between these two health domains across different age groups. Hence, the aim of this study was to determine the risk of eye diseases in relation to age and kidney failure in Taiwanese adults.

## Materials and methods

### Study population

The data used in the current study were obtained from 132,720 individuals who enrolled in the Taiwan Biobank project between 2008 and 2020. These individuals, aged 30–70 years were cancer-free. We excluded blind and color-blind individuals (*n* = 898) and those with missing information (*n* = 4,261). Overall, 127,561 participants were included in the final analysis. The study was approved by the institutional review board of Chung Shan Medical University Hospital (IRB:CS1-20009). All the Biobank participants provided written informed consent during enrollment. All methods were carried out in accordance with relevant guidelines and regulations.

### Definitions of outcome and exposures

Self-reported information on the main outcome, eye diseases (including glaucoma, cataract, xerophthalmia, and retinal detachment) and exposures (kidney failure and age), along with other variables associated with eye diseases such as weight, height, hypertension, hyperlipidemia, smoking, drinking, and exercise, diabetes [8, 11, 12], were obtained from questionnaires designed by Taiwan Biobank. Questions on disease status had two response options: ‘yes’ for having a physician-diagnosed disease and ‘no’ for having no disease. Body mass index (BMI) was calculated as weight in kilograms divided by the square of height in meters and was classified as underweight (under 18.5 kg/m^2^), normal weight (18.5 to 23.9 kg/m^2^), overweight (24 to 26.9 kg/m2), and obese (27 kg/m^2^ or more). Age was grouped into three categories: <50 years, 50–60 years, and ≥ 60 years.

### Statistical analyses

Data were analyzed using the SAS software (version 9.4; SAS Institute, Cary, NC, USA). Descriptive data were analyzed using the Chi-square test and were presented as numbers (n) and percentages (%). Multiple logistic regression was used to explore the association between kidney failure and eye diseases. Covariates in the multiple logistic regression model included age, sex, cigarette smoking, alcohol drinking, exercise, BMI, hypertension, diabetes, and hyperlipidemia. Multiple logistic regression was also used to determine the interaction between age and kidney failure on eye diseases. The results were reported in odds ratio (PORs) and 95% confidence intervals (CIs).

## Results

Table 1 shows the demographic characteristics of the study participants categorized by the presence or absence of kidney failure. A total of 127,561 participants (46,246 men and 81,315 women) were included in the study. Cases with kidney failure and controls significantly differed with respect to glaucoma, cataract, and retinal detachment (*p*-value < 0.05). Moreover, cases and controls differed significantly with respect to sex, age, hypertension, diabetes, and hyperlipidemia (*p*-value < 0.05).

**Table 1:** Demographic attributes of study subjects based on the presence or absence of kidney failure

Variables	No kidney failure	Kidney failure	*P*-value
	(*n* = 127,404)	(*n* = 157)	
Eye diseases, n (%)			< 0.0001
No eye disease	97,113 (76.22)	93 (59.24)	
At least one eye disease	30,291 (23.78)	64 (40.76)	
Glaucoma, n (%)			0.0250
No	125,851 (98.78)	152 (96.82)	
Yes	1553 (1.22)	5 (3.18)	
Cataract, n (%)			< 0.0001
No	117,832 (92.49)	121 (77.07)	
Yes	9572 (7.51)	36 (22.93)	
Xerophthalmia, n (%)			0.2081
No	114,259 (89.68)	136 (86.62)	
Yes	13,145 (10.32)	21 (13.38)	
Retinal detachment, n (%)			< 0.0001
No	125,922 (98.84)	148 (94.27)	
Yes	1482 (1.16)	9 (5.73)	
Floaters, n (%)			0.0098
No	113,009 (88.70)	129 (82.17)	
Yes	14,395 (11.30)	28 (17.83)	
Age, years, n (%)			< 0.0001
Age < 50	61,511 (48.28)	50 (31.85)	
50 ≤ age < 60	37,630 (29.54)	45 (28.66)	
Age ≥ 60	28,263 (22.18)	62 (39.49)	
Sex, n (%)			< 0.0001
Women	81,244 (63.77)	71 (45.22)	
Men	46,160 (36.23)	86 (54.78)	
Cigarette smoking, n (%)			0.7386
No	101,982 (80.05)	124 (78.98)	
Yes	25,422 (19.95)	33 (21.02)	
Alcohol drinking, n (%)			0.2042
No	116,423 (91.38)	139 (88.54)	
Yes	10,981 (8.62)	18 (11.46)	
Exercise, n (%)			0.4545
No	76,758 (60.25)	90 (57.32)	
Yes	50,646 (39.75)	67 (42.68)	
BMI categories, kg/m^2^, n (%)			0.1735
Normal weight (18.5 ≤ BMI < 24)	62,329 (48.92)	67 (42.68)	
Underweight (BMI < 18.5)	4242 (3.33)	8 (5.10)	
Overweight (24 ≤ BMI < 27)	34,444 (27.04)	41 (26.11)	
Obese (BMI ≥ 27)	26,389 (20.71)	41 (26.11)	
Hypertension			< 0.0001
No	112,296 (88.14)	89 (56.69)	
Yes	15,108 (11.86)	68 (43.31)	
Diabetes			< 0.0001
No	115,490 (90.65)	118 (75.16)	
Yes	11,914 (9.35)	39 (24.84)	
Hyperlipidemia			< 0.0001
No	118,153 (92.74)	121 (77.07)	
Yes	9251 (7.26)	36 (22.93)	

Abbreviations: n, sample size; %, percent; BMI, body mass index; kg, kilogram; m^2^, meter squared

Table 2 displays the odds of having at least one eye disease. Compared to non-cases, kidney failure was associated with a higher risk of eye diseases (POR = 1.815; 95% CI = 1.29; 1-2.551). Compared to age < 50 years, older age was also associated with a higher risk of eye diseases. The POR was 2.252 (95% CI = 2.176–2.330) for 50 ≤ age < 60 years and 4.752 (95% CI = 4.580–4.930) for age > 60 years.

**Table 2:** Results of logistic regression showing the odds of having at least one eye disease

Variables	POR	95% CI	*P*-value
Kidney failure			
No (ref.)	-	-	-
Yes	1.815	1.291–2.551	0.0006
Age			
Age < 50 (ref.)	-	-	-
50 ≤ age < 60	2.252	2.176–2.330	< 0.0001
Age ≥ 60	4.752	4.580–4.930	< 0.0001
Sex			
Women (ref.)	-	-	-
Men	0.618	0.597–0.640	< 0.0001
Cigarette smoking			
No (ref.)	-	-	-
Yes	0.876	0.839–0.915	< 0.0001
Alcohol drinking			
No (ref.)	-	-	-
Yes	0.919	0.869–0.972	0.0029
Exercise			
No (ref.)	-	-	-
Yes	1.127	1.096–1.160	< 0.0001
BMI categories			
Normal weight (ref.)	-	-	-
Underweight	1.069	0.991–1.153	0.0850
Overweight	0.869	0.841–0.899	< 0.0001
Obese	0.783	0.754–0.814	< 0.0001
Hypertension			
No (ref.)	-	-	-
Yes	1.090	1.045–1.137	< 0.0001
Diabetes			
No (ref.)	-	-	-
Yes	1.085	1.037–1.136	0.0004
Hyperlipidemia			
No (ref.)	-	-	-
Yes	1.523	1.451–1.599	< 0.0001

POR, prevalence odds ratio; CI, confidence interval; ref., reference; BMI, body mass index

Table 3 shows the association of kidney failure with each eye disease (glaucoma, cataract, retinal detachment, and xerophthalmia). Kidney failure had a significant relationship only with cataract and retinal detachment. That is, compared to no kidney failure (the reference category), kidney failure was associated with a higher risk of cataract (POR = 2.480; 95% CI = 1.635–3.761) and retinal detachment (POR = 3.885; 95% CI = 1.968–7.666). Kidney failure and age had a significant interaction with respect to cataract (*p*-value = 0.0002).

**Table 3:** Association of kidney failure with eye diseases

Variables	Glaucoma		Cataract		Xerophthalmia		Retinal detachment	
	POR	95% CI	POR	95% CI	POR	95% CI	POR	95% CI
Kidney failure								
No (ref.)	-	-	-	-	-	-	-	-
Yes	1.693	0.688–4.167	2.480	1.635–3.761	1.240	0.771–1.995	3.885	1.968–7.666
Age								
Age < 50 (ref.)	-	-	-	-	-	-	-	-
50 ≤ age < 60	2.404	2.01–2.778	6.857	6.239–7.536	2.001	1.909–2.097	1.767	1.548–2.018
Age ≥ 60	4.040	3.495–4.670	28.351	25.878–31.060	2.899	2.756–3.049	2.362	2.055–2.715
Sex								
Women (ref.)	-	-	-	-	-	-	-	-
Men	1.190	1.054–1.344	0.788	0.744–0.835	0.382	0.362–0.403	1.360	1.204–1.535
Cigarette smoking								
No (ref.)	-	-	-	-	-	-	-	-
Yes	0.917	0.790–1.064	1.037	0.967–1.112	0.891	0.834–0.952	0.961	0.831–1.112
Alcohol drinking								
No (ref.)	-	-	-	-	-	-	-	-
Yes	0.925	0.766–1.117	0.956	0.877–1.043	0.967	0.888–1.052	0.899	0.743–1.088
Exercise								
No (ref.)	-	-	-	-	-	-	-	-
Yes	1.084	0.976–1.204	1.138	1.087–1.192	1.098	1.055–1.141	1.057	0.948–1.177
BMI categories								
Normal weight (ref.)	-	-	-	-	-	-	-	-
Underweight	1.070	0.787–1.454	1.321	1.160–1.504	1.138	1.034–1.253	1.265	0.952–1.682
Overweight	0.847	0.748–0.959	0.897	0.851–0.946	0.884	0.845–0.925	0.988	0.874–1.118
Obese	0.928	0.809–1.064	0.853	0.801–0.909	0.777	0.736–0.821	0.907	0.786–1.047
Hypertension								
No (ref.)	-	-	-	-	-	-	-	-
Yes	1.297	1.137–1.481	1.212	1.145–1.283	1.033	0.974–1.094	1.209	1.045–1.398
Diabetes								
No (ref.)	-	-	-	-	-	-	-	-
Yes	1.611	1.409–1.843	1.433	1.349–1.522	0.893	0.837–0.953	1.189	1.017–1.391
Hyperlipidemia								
No (ref.)	-	-	-	-	-	-	-	-
Yes	1.437	1.240–1.665	1.465	1.374–1.563	1.583	1.487–1.686	1.126	0.947–1.339
Age*kidney failure	*P*-value = 0.1268		*P*-value = 0.0002		*P*-value = 0.9102		*P*-value = 0.3535	

POR, prevalence odds ratio; CI, confidence interval; ref., reference; BMI, body mass index; *, the interaction term

Table 4 displays the association between kidney failure and cataract stratified by age groups. Compared to non-cases, kidney failure was significantly associated with a higher risk of cataract among individuals in two age groups; age < 50 years (POR = 6.534; 95% CI = 2.493–17.124) and 50 > age < 60 years (POR = 3.957; 95%CI = 1.986–7.881). Hypertension, diabetes, and hyperlipidemia were significantly associated with a higher risk of cataract in all age groups.

**Table 4:** Association between kidney failure and cataract categorized by age groups

Variables	Age < 50			50 ≤ age < 60			Age ≥ 60		
	POR	95% CI	*P*-value	POR	95% CI	*P*-value	POR	95% CI	*P*-value
kidney failure									
No (ref.)	-	-	-	-	-	-	-	-	-
Yes	6.534	2.493–17.124	0.0001	3.957	1.986–7.881	< 0.0001	1.508	0.880–2.587	0.1353
Sex									
Women (ref.)	-	-	-	-	-	-	-	-	-
Men	1.006	0.827–1.223	0.9551	0.890	0.796–0.995	0.0403	0.725	0.675–0.778	< 0.0001
Cigarette smoking									
No (ref.)	-	-	-	-	-	-	-	-	-
Yes	1.006	0.799–1.265	0.9616	1.027	0.900-1.172	0.6881	1.051	0.962–1.149	0.2682
Alcohol drinking									
No (ref.)	-	-	-	-	-	-	-	-	-
Yes	1.103	0.813–1.496	0.5281	0.877	0.744–1.033	0.1153	0.980	0.880–1.091	0.7077
Exercise									
No (ref.)	-	-	-	-	-	-	-	-	-
Yes	1.223	1.015–1.473	0.0346	1.127	1.037–1.224	0.0046	1.132	1.068-1.200	< 0.0001
BMI categories									
Normal weight (ref.)	-	-	-	-	-	-	-	-	-
Underweight	0.792	0.484–1.296	0.3535	1.332	1.055–1.681	0.0159	1.452	1.226–1.719	< 0.0001
Overweight	1.025	0.830–1.267	0.8183	0.810	0.731–0.896	< 0.0001	0.919	0.861–0.981	0.0116
Obese	0.862	0.684–1.088	0.2114	0.824	0.732–0.927	0.0012	0.843	0.779–0.913	< 0.0001
Hypertension									
No (ref.)	-	-	-	-	-	-	-	-	-
Yes	1.729	1.250–2.392	0.0009	1.277	1.138–1.432	< 0.0001	1.182	1.106–1.263	< 0.0001
Diabetes									
No (ref.)	-	-	-	-	-	-	-	-	-
Yes	2.378	1.765–3.203	< 0.0001	1.515	1.346–1.706	< 0.0001	1.371	1.276–1.473	< 0.0001
Hyperlipidemia									
No (ref.)	-	-	-	-	-	-	-	-	-
Yes	1.820	1.273–2.602	0.0010	1.544	1.362–1.752	< 0.0001	1.409	1.306–1.521	< 0.0001

POR, prevalence odds ratio; CI, confidence interval; ref., reference; BMI, body mass index

Table 5 illustrates the association between age and cataract based on the presence or absence of kidney failure. Compared to the younger age (age < 50 years), older age was significantly associated with a higher risk of cataract in those who had no kidney failure. For 50 ≥ age < 60 years, the OR = 6.895; 95% CI = 6.272–7.581 and for age > 60 years, the POR = 28.606; 95% CI = 26.101–31.352. However, only age > 60 years was significantly associated with cataract among cases with kidney failure (POR = 3.504; 95% CI = 1.040-11.802).

**Table 5:** Association between age and cataract based on the presence or absence of kidney failure

Variables	No kidney failure			kidney failure		
	OR	95% CI	*P*-value	OR	95% CI	*P*-value
Age						
Age < 50 (ref.)	-	-	-	-	-	-
50 ≤ age < 60	6.895	6.272–7.581	< 0.0001	2.258	0.623–8.190	0.2153
Age ≥ 60	28.606	26.101–31.352	< 0.0001	3.504	1.040-11.802	0.0430
Sex						
Women (ref.)	-	-	-	-	-	-
Men	0.788	0.744–0.835	< 0.0001	0.525	0.183–1.508	0.2315
Cigarette smoking						
No (ref.)	-	-	-	-	-	-
Yes	1.040	0.969–1.115	0.2794	0.290	0.071–1.183	0.0844
Alcohol drinking						
No (ref.)	-	-	-	-	-	-
Yes	0.953	0.874–1.039	0.2780	2.067	0.508–8.404	0.3104
Exercise						
No (ref.)	-	-	-	-	-	-
Yes	1.137	1.086–1.191	< 0.0001	1.572	0.627–3.940	0.3343
BMI categories						
Normal weight (ref.)	-	-	-	-	-	-
Underweight	1.317	1.156-1.500	< 0.0001	4.629	0.856–25.040	0.0752
Overweight	0.897	0.851–0.947	< 0.0001	0.746	0.253–2.202	0.5963
Obese	0.856	0.803–0.911	< 0.0001	0.532	0.156–1.812	0.3126
Hypertension						
No (ref.)	-	-	-	-	-	-
Yes	1.210	1.142–1.281	< 0.0001	1.919	0.715–5.152	0.1956
Diabetes						
No (ref.)	-	-	-	-	-	-
Yes	1.427	1.343–1.516	< 0.0001	6.171	2.106–18.084	0.0009
Hyperlipidemia						
No (ref.)	-	-	-	-	-	-
Yes	1.473	1.381–1.571	< 0.0001	0.268	0.076–0.943	0.0402

OR, odds ratio; CI, confidence interval; ref., reference; BMI, body mass index

Table 6 shows the risk of cataract based on a combination of age and the presence or absence of kidney failure. The reference group comprised non-kidney failure individuals aged below 50 years. Compared to the reference group, the risk of cataract was significantly higher in non-kidney failure individuals aged 50–60 (POR = 6.895, 95% CI = 6.272–7.581) and > 60 years (POR = 28.602, 95% CI = 26.098–31.347) as well as in patients with kidney failure aged below 50 (POR = 10.725; 95% CI = 4.227–27.211), 50–60 (POR = 28.487; 95% CI = 14.270-56.866) and above 60 years (POR = 43.183; 95% CI = 24.434–72.824).

**Table 6:** Risk of cataract based on age in combination with the presence or absence of kidney failure

Variables	OR	95% CI	*P*-value
Age < 50, no kidney failure (ref.)	-	-	-
50 ≤ age < 60, no kidney failure	6.895	6.272–7.581	< 0.0001
Age ≥ 60, no kidney failure	28.602	26.098–31.347	< 0.0001
Age < 50, kidney failure	10.725	4.227–27.211	< 0.0001
50 ≤ age < 60, kidney failure	28.487	14.270-56.866	< 0.0001
Age ≥ 60, kidney failure	42.183	24.434–72.824	< 0.0001
Sex			
Women (ref.)	-	-	-
Men	0.788	0.745–0.835	< 0.0001
Cigarette smoking			
No (ref.)	-	-	-
Yes	1.037	0.966–1.112	0.3164
Alcohol drinking			
No (ref.)	-	-	-
Yes	0.957	0.877–1.043	0.3160
Exercise			
No (ref.)	-	-	-
Yes	1.138	1.086–1.191	< 0.0001
BMI categories			
Normal weight (ref.)	-	-	-
Underweight	1.323	1.162–1.506	< 0.0001
Overweight	0.897	0.850–0.946	< 0.0001
Obese	0.854	0.802–0.909	< 0.0001
Hypertension			
No (ref.)	-	-	-
Yes	1.210	1.143–1.281	< 0.0001
Diabetes			
No (ref.)	-	-	-
Yes	1.433	1.349–1.522	< 0.0001
Hyperlipidemia			
No (ref.)	-	-	-
Yes	1.465	1.373–1.562	< 0.0001

OR, odds ratio; CI, confidence interval; ref., reference; BMI, body mass index

Table 7 shows the risk of kidney failure determined by an interplay of age and cataract. Compared to age < 50 years and no cataract (the reference category), the risk of kidney failure was significantly higher among patients with cataract regardless of age. The PORs; 95% CIs were 9.510; 3.722–24.297 for cataract patients aged below 50 years, 4.1109; 2.072–8.145 for the patients aged between 50 and 60 years, and 2.252; 1.264–4.014 for those aged above 60 years.

**Table 7:** Risk of kidney failure determined by an interplay of age and cataract

Variables	OR	95% CI	*P*-value
Age < 50, no cataract (ref.)	-	-	-
50 ≤ Age < 60, no cataract	1.034	0.650–1.646	0.8870
Age ≥ 60, no cataract	1.563	0.978–2.498	0.0618
Age < 50, cataract	9.510	3.722–24.297	< 0.0001
50 ≤ Age < 60, cataract	4.109	2.072–8.145	< 0.0001
Age ≥ 60, cataract	2.252	1.264–4.014	0.0059

OR, odds ratio; CI, confidence interval; ref., reference; BMI, body mass index. Adjusted for sex, smoking, drinking, exercise, BMI, hypertension, diabetes, and hyperlipidemia

## Discussion

Our investigation, centered on Taiwanese adults, unveiled a noteworthy correlation between kidney failure and cataract. Specifically, individuals with kidney failure exhibited a higher cataract risk compared to those without the condition. Similar findings have been reported previously [13, 14]. The potential explanation for this association lies in the shared developmental, structural, and genetic pathways between the kidney and the eye. Both organs may be closely linked in terms of disease pathways, encompassing vascular remodeling, atherosclerosis, inflammation, endothelial dysfunction, and oxidative stress, which are common mechanisms associated with chronic kidney disease (CKD) and various eye diseases [8].

However, contrasting results were observed in another study that found no significant impact of renal function on cataract incidence [15]. The study, however, noted an age-dependent association between worse renal impairment and the likelihood of cataract surgery. In our study, we identified a significant interaction between age and kidney failure with respect to cataract risk. Notably, the risk was higher in younger patients (below 50 years) compared to older individuals (50 years and above), aligning with prior research [15] suggesting elevated odds of cataract surgery (POR, 2.75; CI, 1.06–7.14) in individuals below 60 years with moderate to severe renal impairment. Additionally, the odds ratio diminshed as age increased. These observations could be influenced by several factors. For instance, kidney failure is often associated with chronic conditions such as diabetes and hypertension. These conditions, especially if present at a younger age, may contribute to an increased likelihood of developing cataracts. Chronic kidney disease is known to be associated with accelerated aging [16], affecting various organs, including the eyes. Furthermore, cataracts are typically associated with aging, and this acceleration in aging processes in individuals with renal impairment may contribute to an earlier onset. It is also important to state that some medications used to manage kidney failure or associated conditions may have side effects on ocular health [17], potentially influencing the development of cataracts. Finally, the overall health, genetics, and lifestyle factors of individuals with kidney failure can play a role. For example, individuals with kidney disease may be more prone to oxidative stress, which is implicated in the development of cataracts [18]. Despite these, further research and a deeper exploration of the underlying mechanisms would be needed to provide a comprehensive understanding of these associations.

From a public health perspective, our findings emphasize the importance of younger patients with kidney failure paying attention to their eye health. Some studies have highlighted the importance of eye disease prevention in patients with chronic kidney disease [6, 19]. For instance, a previous study recommended screening patients with CKD for eye diseases, especially cataract which is reversible [6]. A previous study conducted in Taiwan revealed that the risk of cataract might increase with the severity of renal impairment [13]. In our study, the POR for the association of kidney failure with at least one eye disease was 1.815 and 2.480 with cataract. Another study conducted in 2012 found an association between kidney failure and eye diseases among Taiwanese [14]. The study, however, was limited in that lifestyle factors were not taken into consideration. Our study adjusted for lifestyle behaviors, enhancing the credibility of the results.

The notable interplay observed in the present study between age and kidney failure regarding cataract suggests that the extent of cataract severity may be contingent upon an individual’s age and renal health status. The risk of cataract was significantly higher in patients with kidney failure in all age groups. Notably, the highest POR was reported in the youngest age group (< 50 years).

In our study, men had a significantly lower cataract risk than women, possibly linked to differences in estrogen concentrations. [20].. Comorbidities like hypertension, diabetes, and hyperlipidemia were associated with a higher risk of cataract, consistent with previous reports [21].

Overall, the study’s strength lies in its robust methodology, large and diverse sample, comprehensive assessment of eye diseases, and the nuanced exploration of the interrelationships between age, kidney failure, and specific ocular conditions. The current study has some limitations. First, the definition of diseases was based on self-reports, which might have caused some classification errors. Furthermore, the questionnaires did not contain information on other eye conditions such as diabetic retinopathy and age-related macular degeneration alongside others, so, we could not assessed them. Second, the study design was cross-sectional, so, the findings cannot clearly explain causal relationships. Though our study paints a picture of the relationship between eye diseases and kidney failure, large-scale population studies are needed to support the findings.

## Conclusions

In our research, we found that both kidney failure and age were individually linked to a heightened risk of eye diseases. Furthermore, both variables were independently and collaboratively related to an increased risk of cataract. Notably, when considering age, there was a bidirectional connection between cataracts and kidney failure, especially among individuals below 50 years. This bidirectional relationship between kidney failure and cataract in younger adults underscores the importance of screening for kidney failure among younger individuals with cataracts and vice versa. In light of the growing elderly population and the associated rise in age-related health conditions, including eye diseases and kidney failure, this study holds the potential to contribute valuable insights for public health initiatives, clinical management, and future research endeavors.

## Data Availability

The data that support the findings of this study are available from Taiwan Biobank but restrictions apply to the availability of these data, which were used under license for the current study, and so are not publicly available. Data are however available from the corresponding author, Prof. Yung-Po Liaw upon reasonable request and with permission from Taiwan Biobank.
